# Valorisation of Raspberry Seeds in Cosmetic Industry-Green Solutions

**DOI:** 10.3390/pharmaceutics16050606

**Published:** 2024-04-29

**Authors:** Ivanka Ćirić, Dragana Dabić Zagorac, Milica Sredojević, Milica Fotirić Akšić, Biljana Rabrenović, Stevan Blagojević, Maja Natić

**Affiliations:** 1 Innovative Centre Faculty of Chemistry Belgrade, University of Belgrade, Studentski Trg 12-16, 11158 Belgrade, Serbia; ivankai@chem.bg.ac.rs (I.Ć.); ddabic@chem.bg.ac.rs (D.D.Z.); pantelicm@chem.bg.ac.rs (M.S.); 2 Faculty of Agriculture, University of Belgrade, Nemanjina 6, 11080 Belgrade, Serbia; fotiric@agrif.bg.ac.rs (M.F.A.); biljanar@agrif.bg.ac.rs (B.R.); 3 Institute of General and Physical Chemistry, Studentski Trg 12-16, 11158 Belgrade, Serbia; 4 Faculty of Chemistry, University of Belgrade, Studentski Trg 12-16, 11158 Belgrade, Serbia

**Keywords:** proline DES, defatted seed cake, seed oil, bioactive compounds, skincare

## Abstract

The fruit processing industry generates large quantities of by-products well known to be rich in bioactive compounds with numerous nutritional properties and beneficial effects for human health. We developed a strategy to valorise raspberry seeds and obtain valuable ingredients with potential application in cosmetic skincare formulas. Cold press extraction technology was applied to extract oil, and the remaining defatted raspberry seed cake was treated with three proline based deep eutectic solvents (DES) to extract polyphenols. The most potent was proline/citric acid extract, with free and total ellagic acid content (52.4 mg/L and 86.4 mg/L), total phenolic content (TPC, 550.1 mg GAE/L) and radical scavenging activity (RSA, 4742.7 mmol TE/L). After the direct mixing of the extract and after encapsulation with starch as a carrier, the skincare emulsion and microemulsion were characterised by irritation potential (Zein test), transepidermal water loss (TEWL), red blood cell (RBC), and DPPH antioxidant test. The resulting preparations were of improved quality in comparison to the control hand cream, with a low skin irritation effect, lower TEWL, and higher antioxidant potential. This work complies with circular economy principles and green technology standards, and represents the efficient model on how to reuse natural resources through waste minimization.

## 1. Introduction

It is fundamental for the principles of the circular economy to be based on the fact that waste is not produced, but rather generated and then disposed of. These principles are essential for waste management and for the sustainable economy of the future. In order to encourage and promote such models locally, but also globally, large funds are being allocated in order to support the implementation of innovative solutions with the aim of utilizing waste. However, certain industries, such as the agri-food sector, produce a huge amount of unavoidable waste material, which in most cases, can be reused [1]. The production of fruit juices, for example, generates a large amount of waste that has the potential for further utilization as biomaterial.

Berry fruits are fruits with outstanding taste and aroma, and are consumed as table fruit, but a large quantity are processed. These are reservoir of minerals, sugars, organic acids, vitamins, carotenoids, phenols, fatty acids, and others [2,3,4]. Raspberries are highly valued berry fruits cultivated all over the world for their delicious taste. In the year 2022, the annual production of raspberries in Europe was over 616 thousand tons. Serbia was ranked as the second country in Europe in terms of production with over 116,000 tons [5]. The consumable part of the raspberry plant is the main source of energy and nutrients, whereas the non-consumable parts are source of residues that are considered as agricultural waste. Due to the high content of bioactive compounds, especially ellagic acid, raspberries are considered antimutagenic, antimicrobial, anticancer, antidiabetic, anti-inflammatory, antioxidant, and neuro and cardioprotective properties [6].

The most attractive means of waste management among chemists is to consider efficient extraction methods to reuse waste and residues. The new trend, widely explored in finding new resources of raw materials is to apply solvents composed of naturally occurring ingredient. Green extraction applying deep eutectic solvents is promising technology largely discussed in literature at present [7,8].

This work deals with the possible utilization of seed material generated in the industrial processing of raspberry juice, known to be an excellent source of ingredients with antioxidant and antimicrobial potential. Our intent was to address the possible reuse of this valuable and non-toxic secondary raw material, in accordance with good production practices and legal regulations in the cosmetic industry [1,9]. Relying on data from the scientific literature, the advantages of the valorisation of fruit and vegetable waste in conventional and personalized cosmetics are obvious. Oils extracted from the seeds are often studied in the cosmetics industry for the production of various preparations and sunscreen formulations [10,11]. Raspberry seed oil is characterized by a high content of polyunsaturated fatty acids (PUFAs), such as linoleic acid and alpha-linolenic acid and tocopherol [12]. As a rich source of vitamins A and E, raspberry seed oil is also used in cosmetics as an effective moisturizer and emollient that helps reduce oxidative stress in the skin and in cosmetic emulsions as UV protection. In addition, these vitamins are necessary for the maintenance and repair of skin cells. Raspberry seed oil can be used as a make-up base [13]. It forms a fine lipid barrier that prevents the skin from losing its natural moisture. All these benefits of raspberry seed oil in skin care have led to the registration of numerous patents, including in China (17 patents) and in the USA (11 patents) [14].

It should be mentioned that the extraction process of oil from seeds generates certain wastes for which reduction and recovery strategies need to be developed. Keeping in mind that roughly 9% of the oil is extracted by cold pressing, almost all of the starting biomass is still wasted. Remaining defatted raspberry seed cake is a rich source of bioactive compounds [12,15,16,17]. Bioactive phytochemicals from berries’ seed oil processing by-products were reported for its chemical richness, heterogeneity, biological, and functional properties [18]. To date, the polyphenolic profile of defatted raspberry seeds was investigated, mainly to determine the bioactivity potential of extracts [19,20]. Marić and coauthors showed that defatted raspberry seeds extract possess high antioxidative, and high antiproliferative activities but low antimicrobial activity, and recommended that they be used in food, cosmetic, and pharmaceutical industries [19]. In addition, in a study conducted by Wójciak and coworkers, it was shown that defatted strawberry seeds are a rich reservoir of phenolic compounds with significant antioxidant properties (tiliroside, kaempferol glucoside, and ellagic acid), and may serve as a valuable material for obtaining a beneficial additive for skin care products [16].

All this was an incentive for us to search for environmentally friendly solutions that could prevent the disposal of defatted raspberry seeds in the landfill, and at the same time support innovations in the field of cosmetics with such a treasure. To this aim, after the isolation of the oil, defatted raspberry seed cake was a starting raw material, and it was used to extract bioactive components using natural deep eutectic solvents (DES). Proline, as bond acceptor and several compounds acting as H-bond donors were selected as components of mixtures. This part of the experiment included (i) the chemical characterization of the cold pressed oil; (ii) the optimization of the green extraction procedure of bioactive components from defatted seed cake; (iii) the determination of the total phenolic content (TPC) and radical scavenging activity (RSA) in the extracts; (iv) the determination of free ellagic acid and total ellagic acid in the extracts by liquid chromatography; and (v) the selection of the most efficient proline-based DES system for the extraction of phenolic compounds.

After selecting the most prominent extract, the experiment was designed to obtain cosmetic formulation and to assess its characteristics. Two products were prepared: emulsion by the direct mixing of the selected extract into the control sample (hand care cream); and microemulsion by encapsulation with starch as a carrier. To evaluate the improvement of skin care formulation, several tests were selected and performed: the Zein test of irritation potential (harshness); transepidermal water loss (TEWL); red blood cell test (RBC); and DPPH antioxidant assay.

## 2. Materials and Methods

### 2.1. Chemicals

All solvents and chemicals used in experiments were of analytical purity grade. Proline, citric, and malic acid were purchased from Thermo Fisher scientific from Acros Organics BV (Geel, Belgium). Thermo Fisher TKA MicroPure water purification system was used for obtaining ultrapure water (0.055 µS/cm) for preparing aqueous solutions of blanks and standards. The SupelcoTM 37 Component FAME standard mix (Bellefonte, PA, USA) was used to identify fatty acid methyl esters. Zea mays protein, sodium dodecyl sulphate (SDS), isopropyl myristate, standards of α-, β-, γ-, and δ-tocopherols were purchased from Sigma-Aldrich (Burghausen, Germany). Syringe filters (13 mm, PTFE membrane 0.45 μm) were purchased from Supelco (Bellefonte, PA, USA).

### 2.2. Seed Oil Cold-Pressing and Defatted Seed Cake Preparation

Raspberry seeds were obtained from a small juice producer in Serbia, as a waste generated during production. The seeds were frozen transported to the laboratory, where they were thawed, washed from pulp residue, and then dried at a temperature of 50 °C to a moisture content of 6%.

The process of the cold pressing of raspberry seeds was carried out with a screw press “Ulimac” (Turkey) with a capacity of 25 kg/h. The temperature of the oil during the pressing process did not exceed 45 °C, which was monitored with a digital thermometer. The oil was stored in plastic cuvettes at a temperature of −20 °C until analysis to assess fatty acid composition, tocopherols, and oxidative stability test.

The defatted seed cake was grounded on a Grindomix GM200 (Retsch, Haan, Germany), sieved on a sieve with a pore size of 250 μm (Retsch, Germany), and stored in closed polyethylene bags at room temperature until extraction of polyphenols.

### 2.3. Determination of the Fatty Acids Composition

The fatty acid methyl esters were prepared and detected according to the standard methods [21,22]. Gas chromatography was used to separate and detect the methyl esters on a gas chromatograph (Agilent Technologies 6890, Lexington, MA, USA) with a split–splitless injector, a flame ionization detector (FID), and a Supelco SP-2560 capillary column (100 m length × 0.25 mm inner diameter × 0.20 μm film thickness, Supelco, Bellefonte, PA, USA) [22]. Helium with a flow rate of 5 mL/min was used as the mobile phase. The injector and detector temperatures were 250 °C and 260 °C, respectively. The injection volume was 1 μL, and the injector distribution ratio was set to 20:1. The column temperature was programmed from an initial 50 °C (held for 5 min)–240 °C (held for 20 min), with a linear temperature change of 4 °C/min. Chromatographic peaks in the sample were identified by comparing the relative retention times of the fatty acid methyl esters from the samples with the Supelco 37 Component FAME Mix Standard. The fatty acid content was calculated in mg/g lipids and expressed in relative amount as a mass percent of total fatty acids. All measurements were performed in a triplicate and results are expressed as mean ± standard deviation.

### 2.4. Determination of the Tocopherol Content

The tocopherol content of oil was determined according to a procedure reported by Gimeno and coworkers [23]. The individual tocopherol isomers were analysed by normal phase HPLC. The column was an XBridge C18 (4.6 × 150 mm, 3.5 µm particle size). The analytical separation of the tocopherol isomers was achieved with an isocratic elution of methanol–water (96:4, *v*/*v*). The injection volume was 20 µL. The total run time and flow rate were 5 min and 2.0 mL/min, respectively. Detection was performed with a DAD detector at 292 nm and each run lasted 6 min.

The oil sample was diluted in hexane (1:10). Then, 200 µL was transferred to a screw cap tube where 800 µL methanol was added. After vortex mixing and centrifugation (3000× *g*, 5 min), the sample was filtered through a 0.22 µm syringe philtre. For each tocopherol standard, an external calibration curve was generated to calculate the amount of tocopherols present in the oil sample [24].

### 2.5. Oxidative Stability Test

The oxidative stability of raspberry seed oil was tested with the OXITEST apparatus (Velp^®^ Scientifica, Usmate, Italy). Oil samples were weighed in two thermostatic titanium chambers of the device (10 g per each). The chambers were hermetically sealed. The temperature was set to 110 °C and the initial O_2_ pressure to 600 kPa. The software (OXISoft, Velp, Italy) was used to monitor the pressure change in the chambers and thus indirectly measure the amount of oxygen consumed by the activity of each component. At the end of the test, the software calculated the induction period (IP) in minutes or hours for each chamber. The longer the IP, the better the oxidative stability of the sample.

### 2.6. DES Extraction of Phenolic Compounds from Defatted Seed Cake

Modified procedure published in the literature was applied for the extraction of phenolic compounds from defatted seed cake [20]. The basic modification of the experiment procedure is the use of proline as a proton acceptor, instead of betaine and sugars. In addition, the preparation conditions, temperature, mixing time and water content were modified and optimized. Solvent was prepared by mixing proline with sucrose in 1:2 molar ratio, or with malic and citric acid in a 1:1 molar ratio. Mixture was heated to 55 °C on a magnetic stirrer for 30 min, until a homogeneous transparent liquid was formed. Obtained solvent was diluted with water (content of water was 30% (*w*/*w*)), and heating with stirring was continued for 45 min. Freshly prepared DES were used for extraction of polyphenols from raspberry seeds press cake. Solid-to-liquid ratio used was 1 g to 10 mL of solvent. Reaction conditions were 30 min at 55 °C on magnetic stirrer. The extract was centrifuged at 10,000 rpm for 10 min, and after the centrifugation was completed, the supernatant was separated. Supernatants were purified using solid–liquid extraction (Solid-Phase Extraction, SPE) under vacuum, on silica gel columns modified with octadecyl-hydrocarbon series (RP-C18 silica gel—MACHERY-NAGEL, Düren, Germany). The column was conditioned with 5 mL of methanol and 5 mL of water. A sample volume of 1 mL was applied to the column, than washed with 10 mL of water, and phenolic compounds were eluted with 5 mL of methanol. The resulting supernatant was stored at 4 °C until further analysis.

### 2.7. Determination of Total Phenolic Content (TPC) and Radical Scavenging Activity (RSA)

TPC and RSA tests were performed on a UV–VIS spectrophotometer (GBC UV-Visible Cintra 6) using procedures described in earlier publication [25]. Before spectrophotometric determinations, all extracts were filtered through 0.45 μm PTFE membrane filters. All measurements were performed in triplicate, and the results are presented as mean ± standard deviation. The Folin–Ciocalteu reagent was used to determine the total phenolic content (TPC). The absorbance of the prepared reaction mixtures was measured at a wavelength of 765 nm, and gallic acid was used as a standard in concentrations of 20, 40, 60, 80, and 100 ppm. The results are expressed as mg of gallic acid equivalents (GAE) per L. The antioxidant activity of the extracts was determined using the DPPH˙ reagent, by measuring the absorbance at 517 nm. A series of standard trolox solutions with concentrations of 100, 200, 300, 400, 500 and 600 μM were prepared. Results are expressed as mmol trolox equivalents (TE) per L.

### 2.8. Determination of Free and Total Ellagic Acid Content

Amounts of total and free ellagic acid in raspberry seed extracts were determined using HPLC-UV. Conditions of HPLC-UV detection were as previously described in [26]. Quantification of ellagic acid was performed using the calibration curve of the ellagic acid standard, within a concentration range of 10−50 ppm. The EA stock solution (200 ppm) was prepared in DMSO. The standard series were prepared by diluting the stock solution so the solvent ratio DMSO/MeOH:H_2_O (1:1) was 25/75 (*v*/*v*). For free ellagic acid determination, extracts after SPE were filtered with 0.45 μm PTFE membrane filters. For total ellagic acid content, extracts were first subjected to acid hydrolysis with hydrochloric acid. The procedure for hydrolysis was slightly modified compared to the one published in the previous paper [26]. To the aliquot (4 mL) of the extract, hydrochloric acid (1.67 mL of 37% HCl, final HCl concentration = 4 M) was added, after which the volume was adjusted to 10 mL adding methanol. The prepared mixture was refluxed for 6 h at 85 °C. After reflux was completed, the sample was brought to the initial volume (10 mL) with methanol. An aliquot of 2 mL was adjusted to pH 2.5 with 5 M NaOH and diluted to 10 mL with methanol. Before HPLC analysis, all extracts were filtered.

### 2.9. Preparation of Cosmetic Formulation

Cosmetic product, the hand care cream from market, additionally declared as a fast-absorbing cream, served as control sample (CS), and was tested after the addition of defatted raspberry seed DES extract. Hand care cream served as control sample, and was used to prepare samples by addition of proline/citric DES extract (0.10, 0.25 and 0.5%) by direct mixing in the cream, and as an encapsulated microemulsion, after mixing with starch as a carrier. Starch is a common additive for the encapsulation of natural oils and extracts; because it is non-toxic, there are no restrictions on its addition to cosmetic products, it is reasonably priced, and it is accepted as an additive for cosmetic products on the INCI list. Due to its structure, starch is suitable for the encapsulation of oils and plant extracts in both O/W and W/O emulsion conditions, and it is very stable and provides a timed release of the active substance.

### 2.10. Investigation of Cosmetic Formulation

The following tests were performed to evaluate the improvement in the properties of the cream: Zein test irritability, TEWL (transepidermal water loss), RBC test (red blood cell test), and antioxidant activity of the sample.

#### 2.10.1. Zein Test of Irritability

For determination of the irritation potential, the Zein test of irritability based on the published Invittox Protocol No. 26. [27,28] was performed. In this in vitro method, 1 g of Zea protein was mixed with the aqueous solution of investigated samples in concentrations of 10 g/L. The determination of dissolved protein was performed according to the Kjeldahl method by measuring the nitrogen content in the aqueous solution. The results of test are expressed as milligrams of nitrogen in 100 mL of sample solution (Zein numbers). Cosmetic products with Zein number values above 400 mg N/100 mL are considered as strongly irritant to the skin, and values in the range 200–400 mg N/100 mL are moderately irritant, whereas the products with very low irritability have results below 200 mg N/100 mL [29].

#### 2.10.2. TEWL (Transepidermal Water Loss)/Hydration

Transepidermal water loss was measured using condensed type closed chamber Aquaflux Biox System Ltd., London, UK. The measurements were performed according to the procedure reported in the literature [30]. For the calculation of the flux density using diffusion-gradient method, the humidity gradient was necessary. The gradient was calculated from two humidity values at two spatially separated points. One value is calculated from the readings of the relative humidity (RH) and temperature (T) sensors mounted in the chamber wall. The other value comes from the condenser, where the humidity can be calculated from its temperature without the need for a second humidity sensor. Obtained results are expressed as flux density (grams of water per square meter per hour).

#### 2.10.3. RBC (Red Blood Cell)/Luminance (L/D) Assay

The RBC test is based on Invittox Protocol No. 37 and was performed according to the procedure described by Petrov Ivanković and coworkers [27]. The red blood cells (RBCs) were isolated by the centrifugation of citrated blood samples obtained from rats. The aliquots of RBC suspension and the test samples were dissolved in PBS pH 7.4 and incubated for 10 min at room temperature. Following incubation, the tubes were centrifuged (1 min at 9000 rpm). SDS was used as internal standard. As an indicator of RBC damage, haemoglobin release was quantified photometrically using Thermo Scientific Evolution 600 spectrophotometer. The absorbance was measured at wavelength range from 450 to 700 nm. The lower concentration of haemoglobin in the formulation has lower irritation potential and better dermatological compatibility.

#### 2.10.4. Antioxidant Activity (DPPH Assay)

Antioxidant value of samples was assessed according to a method published earlier [31]. The method is based on the DPPH reaction of a reagent that has a maximum absorption at 515–520 nm. Three microliters of DPPH solution (0.1 mM prepared in ethanol) was mixed with 1 mL of cream solution (1% *w*/*v* concentration in ethanol). The absorbance was measured after 30 min on 517 nm. Ascorbic acid (10 mg/mL DMSO) was used as the reference and the results were expressed as mmol ascorbic acid equivalents (AAE) per L of the sample.

## 3. Results and Discussion

### 3.1. Characterization of the Raspberry Seed Oil

#### 3.1.1. Fatty Acid Composition

Table 1 shows the composition and content of fatty acids in the raspberry seed oil studied. As can be seen from Table 1, the dominant fatty acid in raspberry seed oil is linoleic acid (ω-6 fatty acid) with a content of 54.80%. This is followed by the monounsaturated fatty acid oleic acid with a content of 11.64% and the α-linolenic (ω-3 fatty acid) with a content of 29.98%. Among the saturated fatty acids, palmitic acid (2.49%) and stearic acid (0.91%) were highlighted. The results show that polyunsaturated essential fatty acids dominate in raspberry seed oil, which is very important from a nutritional point of view, especially with regard to the prevention of cardiovascular diseases and the treatment of metabolic syndrome [32]. However, the high PUFA content significantly affects the oxidative stability of the oil. In particular, the high content of ω-3 fatty acids contributes to its wider application, for example, for non-food purposes, as a component of cosmetic products or in pharmaceutical preparations [33]. Simard and coauthors demonstrated in their study that polyunsaturated fatty acids (PUFAs) play an important role in the establishment and maintenance of the skin barrier function [34]. Previous research has shown linoleic acid, the most common acid in the human epidermis, is directly related to the synthesis of ceramides, which play an important role as a protective layer that prevents moisture loss from the skin, i.e., it influences skin permeability [35,36]. On the other hand, α-linolenic acid, which is very important for skin homeostasis as it is a precursor of long-chain ω-3 PUFAs that produce eicosanoids and resolvins, has never been shown to affect skin permeability [37]. Simard and coauthors proved that, in addition to the individual role of each of the PUFAs mentioned in skin barrier function, their combination is crucial for optimal incorporation and metabolization by the reconstructed tissue [34]. It is proven that the optimal ratio of ω-3 to ω-6 fatty acids should be between 1:1 and 1:4 [38]. As the investigated raspberry seed oil contains about 85% polyunsaturated fatty acid with a ratio of 1:1.8 of essential fatty acids ω-3 and ω-6, it could be an ideal ingredient for cosmetic products.

There is growing evidence that the oral intake of PUFAs, as well as the topical application of PUFA-containing products, improves skin barrier function and vitality, leading to relief of various skin conditions associated with inflammation (e.g., photoaging, atopic dermatitis, or psoriasis) [39]. Cosgrove and coauthors conducted a comprehensive study of 4025 women aged 40–74 years and investigated the relationship between nutrient intake and skin aging, defined as wrinkled appearance, senile dryness, and skin atrophy [40]. One of the conclusions was that a higher intake of linoleic acid was associated with a lower likelihood of senile dryness and skin atrophy.

#### 3.1.2. Tocopherols Content

The composition and content of the individual tocopherol isomers are shown in Table 1. The predominant tocopherol isomer in the oil analysed was γ-tocopherol with a content of 200.39 mg/100 g oil. This was followed by α-tocopherol (69.26 mg/100 g) and δ-tocopherol (28.82 mg/100 g), while β-tocopherol was not detected in this oil. The results obtained were in agreement with the literature data [41,42]. Although α-tocopherol has long been considered to have the most potent antioxidant properties in vivo, studies that are more recent have shown that both γ- and δ-tocopherol have significant antioxidant and anti-inflammatory properties that are superior to α-tocopherol in the prevention and treatment of chronic diseases [43]. Nevertheless, α-tocopherol (vitamin E) is a superior antioxidant in the anti-ageing industry. It helps to protect cells from oxidative damage and maintain collagen structure [14]. Several studies have demonstrated the synergistic effect of vitamin E and vitamin C in terms of photoprotection [44,45]. The oral intake of vitamin E is recommended for many skin therapies, such as yellow nail syndrome, epidermolysis bullosa, cutaneous ulcers, pressure ulcers and burns, sub corneal pustular dermatosis, scleroderma, morphea, calcinosis cutis, Raynaud’s phenomenon, and inflammatory diseases [46]. These conclusions indicate that the studied oil, rich in γ-tocopherol and α-tocopherol, is nutritionally extremely valuable, and the high content of these isomers certainly extends its potential applications for pharmaceutical and cosmetic purposes.

#### 3.1.3. Oxidative Stability

Table 1 also shows the value for the induction period (IP) of the tested cold-pressed raspberry seed oil, IP = 8.3 h. As already stated, the oxidative stability of the oil was tested with the OXITEST (Velp, Italy), which is relatively new on the market in comparison to the Rancimat (Metrohm, Switzerland), and has only been in use for a short time. Thereof, there are scarce data in the literature, especially for this kind of oil. Recently, Tsao and coworkers published results of the oxidative stability using an OXITEST [47]. The investigation included 10 samples of cold-pressed oils of almonds, black sesame, white sesame, camellia seeds, golden linseed, peanuts, pecans, pine seeds, pumpkin seeds, sunflower seeds, and walnuts. The IP (h) of the tested samples at 100 °C and a pressure of 600 kPa ranged from 1.78 h (golden linseed oil) to 33.62 h (peanut oil). For this reason, our result was only comparable with the data reported by Parry and coauthors, who investigated the oxidative stability of cold-pressed raspberry seed oil using the Rancimat test, in which the IP was 20.3 h [12]. Considering that Tinello and coauthors found a strong linear correlation between the results obtained with OXITEST and Rancimat, with the results obtained with OXITEST being about two times lower than those obtained with Rancimat, it can be said that the value obtained in this study is consistent with this [48]. We find the OXITEST as a very good alternative to Rancimat as it takes less time and requires no special sample preparation.

### 3.2. DES Extraction of Phenolic Compounds from Defatted Seed Cake

Defatted seed cake was used for the extraction of polyphenols with deep eutectic solvents. For the preparation of deep eutectic solvents like proline, amino acid was chosen as hydrogen bond acceptor because of its suitability for cosmetic preparations (INCI list). Organic acids (malic and citric acid) and sucrose were chosen as hydrogen bond donors. Proline and all of the solvents are safe additives in cosmetic products. Proline is used as a moisturizing ingredient; sucrose and malic acid are natural humectant, while citric acid serves to preserve cosmetics and personal care products by chelating metal ions. Our intention was to determine the most suitable proline based deep eutectic solvent for extraction of ellagic acid and other phenolic compounds present in defatted raspberry seeds. Therefore, three extracts were compared in terms of antioxidant activity, total phenolic content, and free and total ellagic acid content (Table 2).

#### 3.2.1. Total Phenolic Content (TPC) and Radical Scavenging Activity (RSA)

TPC and RSA were studied in three NADES extracts composed of proline/sucrose, proline/malic acid, and proline/citric acid and results are presented in Table 2. DES extract containing proline and citric acid revealed the highest TPC value, amounting 550.1 mg GAE/L. As for the DPPH assay, the highest value for antioxidant activity was obtained for DES extract where proline and sucrose were in 1:2 molar ratio (5467.5 mmol TE/L). The lowest values for both parameters, TPC (438.1 mg GAE/L) and RSA (3959.9 mmol TE/L), were obtained when the combination of proline and malic acid was used for the extraction of bioactiove compounds.

Published results concerning the total phenolic content and radical scavenging activity of defatted raspberry seeds are scarce, so the comparison of the results was not easy task. However, we refer to the results that we came across, and that we consider suitable for commenting as the most similar to our research. Qin and coauthors published data on the total phenolic content of undigested raspberry seeds extract, and only 320.4 mg GAE/100 g dry matter was found [49]. In a study by Teslić and coworkers, the supercritical extraction with CO_2_ as solvent was applied to obtain defatted raspberry seeds [20]. Different combinations of DES composed of organic acids and betaine or sugars in different molar ratios were prepared to extract phenolic compounds. The total phenolic content of defatted raspberry seeds extracts was in the range of 26.91–39.80 mg GAE/g of dry matter [20]. In order to make the utilization of the fruit wastes more sustainable, Vázquez-González and coworkers studied the extraction of bioactive compounds from raspberry extrudes using choline chloride and betaine-based DES [50]. The obtained results of the seven DES extracts revealed TPC and RSA in ranges between 1838–3356 µg GAE/g and 0.70–1.70 mmol TE/kg of fresh weight, respectively. All the data indicate raspberry by-products as valuable sources of the bioactive compounds, so raspberry defatted seeds could be considered a good basis for further investigations.

#### 3.2.2. Determination of Free and Total Ellagic Acid Content

**Figure 1:**
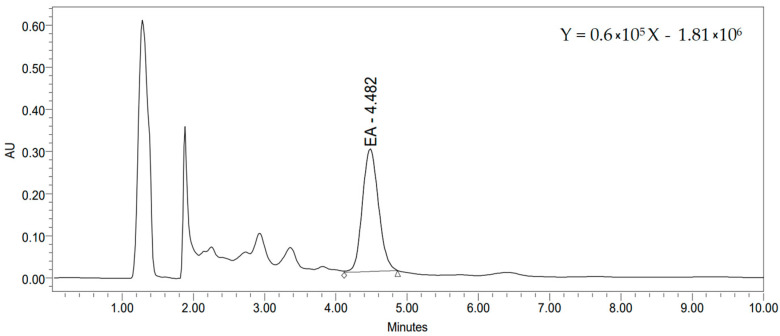
Chromatogram of the proline/citric acid extract of defatted raspberry seed.

Due to its various biological effects, ellagic acid is considered as the most important phenolic compound found in raspberry seeds. Some of the most investigated activities of ellagic acid were antioxidant, anti-carcinogenic, anti-obesity, anti-inflammatory, and anti-angiogenic, anti-neurodegenerative, hepatoprotective activity [51,52]. EA has the ability to scavenge free radicals and, therefore, reduce oxidative stress, which promotes the occurrence of various diseases. Free ellagic acid content determined in the DES extract of defatted raspberry seeds was in the range of 25.2–52.4 mg/L, while the total ellagic acid content was found to be from 66.3 to 86.5 mg/L (Table 2). The highest free and total content of EA was found in proline/citric acid extract (52.4 and 86.5 mg/L, respectively). Figure 1 shows a chromatogram of the proline/citric acid extract of defatted raspberry seed and regression equation (*R*^2^ = 0.997).

The results of our work are in agreement with the results found in the literature. In a study conducted by Teslić and coworkers, the hydrolysis of ellagitanins and extraction of ellagic acid from defatted raspberry seeds were optimized with acidic NADES [20]. Extract with citric acid/betaine/water with molar ratio 2:1:2 exhibited the highest concentration of EA (3.20 mg/100 g extract), while extracts with tartaric acid/betaine and malic acid/betaine (both with molar ratio 2:1) showed the content of 3.18 mg/100 g extract and 3.01 mg/100 g extract, respectively. Also, Marić and coauthors prepared extracts by using an ultrasound-assisted extraction technique, and found a free EA content from 43.05 mg/100 g to 46.76 mg/100 g extracts depending of raspberry variety [19]. The content of free ellagic acid in our extracts is comparable with results from both publications.

However, some other results were not comparable with our work, where extraction was performed with organic solvents, such as the investigation of undigested raspberry seeds, where the extraction solvent was 70% EtOH, and revealed 513.5 mg EA/100 g dry matter [49]. These values are about 10 times higher than our values, and the reason is due to the use of an organic solvent and different extraction procedure.

The most prominent extract was selected based on the highest content of the free ellagic acid and total ellagic acid. In addition, the total phenolic content (TPC) and radical scavenging activity (RSA) were important parameters of the overall antioxidants and polyphenols, and accordingly proline/citric acid was chosen to be incorporated in cosmetic formulation. Emulsion was prepared by directly mixing the selected extract into the control cream sample, and the microemulsion was prepared by encapsulation with starch as a carrier.

### 3.3. Characterization of Cosmetic Formulation

In Table 3, the results of the tests performed in order to assess characteristics of the hand care cream formulations after adding functional raspberry seed extract in different concentrations are presented. The proline/citric acid extract of the defatted seed served as a functional additive for preparing enriched control sample and starch-encapsulated microemulsion. Extracts were added in concentrations of 0.1%, 0.25%, and 0.5%, and tests were performed to obtain an insight into the characteristics of the samples and whether there is an improvement with increasing concentrations. Both experiments led us to conclude that incorporating the extract has a positive effect on the properties of the cosmetic formulation.

Our results showed that the Zein number decreases with an increasing concentration of added extracts in both experiments. The control cream sample had a Zein number of 18, while the cream sample with 0.5% extract and 0.5% starch-encapsulated extract showed significantly lower or no irritancy described by the Zein number values of 10 and 5, respectively. The irritation potential is directly proportional to the quantity of dissolved proteins. Decreasing values of Zein number indicates an improvement in the product’s ability to retain moisture and form a protective layer on the skin. The results of the TEWL test were also concentration-dependent, showing an enhanced hydration effect for all tested concentrations in comparison to the control cream sample. The TEWL value decreased with increasing the percent of added DES extract. Similarly, in starch microemulsions, the values of TEWL decreased with the increasing percent of the DES extract, suggesting better skin hydration. According to the results of the RBC test, adding proline DES raspberry seed extract led to dermatological compatibility increase—the RBC parameter increases with higher concentrations of extract and with starch encapsulation. In addition, antioxidant activity increases with an increasing concentration of DES extract and with starch encapsulation. This may indicate an increase in antioxidant activity and a reduction in skin damage caused by free radicals. This is important for protecting skin from oxidative stress and preventing premature aging.

Overall, adding defatted raspberry seed proline/citric acid extracts into the control sample has a positive impact on the properties of the hand care cream, especially in terms of hydration, antioxidant activity, and skin protection. Such results are expected and are directly connected with the presence of ellagic acid and other polyphenols in the extract.

## 4. Conclusions

There is no doubt that the public’s rising interest in functional ingredients and new, pro-health products has brought searching for new sources of bioactive compounds from raw materials into the spotlight. To this date, defatted seed biomass has not been fully explored and therefore it is underutilized. Defatted seed press cake contains large amounts of dietary fibres, proteins, minerals, and bioactive compounds. Its full potential could be used in a better way if there were more studies, especially if they refer to unknown or overlooked ingredients. Chemical studies that are more detailed are necessary to elucidate the composition of the berry seed oil processing by-products, and its value in the future.

This paper demonstrates in practice the possibilities of a circular approach in the field of the utilization of wasted biomass. Investigation was set in the frame of broader research that is directed at solving environmental problems, primarily the amount of waste generated in the agri-food industry. We proposed a series of steps whose implementation should provide an opportunity for any business entity to develop its own production responsibly, based on the principles of the circular economy, while preserving the environment. The whole approach has potential for implementation on larger scale, from laboratory to industry. The commercialization of a cosmetic formulation could have a direct impact on the efficiency of the use of raw materials, contributing to sustainable development, especially if the cooperation of subjects from the food industry, which generates a large amount of organic waste, and the cosmetic industry is established.

The results presented in this paper suggest that it is possible to achieve some improvements in cosmetic products, which justifies the use of raspberry seeds. The entire procedure is carried out using safe non-toxic solvents, and therefore, it is possible that residue after the extraction of oil and the extraction of polyphenols could be used for products with exfoliating effect.

## Figures and Tables

**Table 1:** Characterization of cold pressed raspberry seed oil.

Fatty acids (%)	
Palmitic C16:0	2.49 ± 0.20
Heptadecanoic acid C17:0	0.06 ± 0.02
Stearic C18:0	0.91 ± 0.15
Oleic C18:1	11.64 ± 0.90
Linoleic C20:2	54.80 ± 1.23
α-Linolenic C20:3	29.98 ± 0.77
Arachidic C20:0	0.04 ± 0.01
Gondoic C20:1	0.06 ± 0.01
ΣSFA *	7.00 ± 0.38
ΣMUFA	11.70 ± 0.96
ΣPUFA	84.78 ± 2.00
Induction period (IP) (h)	8.30 ± 1.16
Tocopherols (mg/100 g)	
α-tocopherol	69.26
β-tocopherol	n.d.
γ-tocopherol	200.39
δ-tocopherol	28.82

The results represent the mean ± standard deviation of the analysis performed in triplicate. * ΣSFA—saturated fatty acid; ΣMUFA—monounsaturated fatty acid; ΣPUFA—polyunsaturated fatty acid.

**Table 2:** Content of TPC, RSA, and free and total ellagic acid.

DES	TPC (mg GAE/L)	RSA (mmol TE/L)	Free Ellagic Acid (mg/L)	Total Ellagic Acid (mg/L)
proline/sucrose	477.6 ± 0.71	5467.5 ± 19.86	25.2 ± 0.1	66.3 ± 0.1
proline/malic acid	438.1 ± 3.53	3959.9 ± 46.35	35.4 ± 0.4	75.7 ± 0.2
proline/citric acid	550.1 ± 3.76	4727.7 ± 39.73	52.4 ± 0.1	86.4 ± 0.2

**Table 3:** Test results of hand care cream control sample (CS) before and after the addition of the proline/citric acid extract.

Sample	Zein Number (mg/100 mL)	TEWL (g/m^2^ h)	RBC (L/D)	Antioxidant Activity DPPH Assay (mmol AAE/L)
Control sample (CS)	18 ± 1	13.2 ± 0.6	22 ± 2	60 ± 3
CS + 0.1% extract	15 ± 1	11.1 ± 0.5	50 ± 4	85 ± 4
CS + 0.25% extract	12.0 ± 0.5	10.2 ± 0.5	103 ± 5	96 ± 4
CS + 0.50% extract	10.0 ± 0.5	9.9 ± 0.5	110 ± 5	115 ± 4
CS + 0.1% starch-encapsulated extract	8.0 ± 0.4	10.2 ± 0.4	115 ± 5	108 ± 4
CS + 0.25% starch-encapsulated extract	6.0 ± 0.3	9.8 ± 0.4	125 ± 5	115 ± 4
CS + 0.50% starch-encapsulated extract	5.0 ± 0.3	9.0 ± 0.4	137 ± 6	126 ± 5

## Data Availability

Data are contained within the article.
